# Supplementary material for: Chromosome-autonomous feedback down-regulates meiotic DNA break competence upon synaptonemal complex formation
Source: Genes Dev. 2020 Dec 1;34(23-24):1605–18. doi: 10.1101/gad.342873.120 (PMC7706706; doi:10.1101/gad.342873.120)
Supplement: Supplemental Material [file supp_34_23-24_1605__index.html]

Chromosome-autonomous feedback down-regulates meiotic DNA break competence upon synaptonemal complex formation — Supplemental Material 

# Chromosome-autonomous feedback down-regulates meiotic DNA break competence upon synaptonemal complex formation

## Supplemental Material

- Supplemental\_material.pdf
