# Supplementary material for: Chromosome-autonomous feedback down-regulates meiotic DNA break competence upon synaptonemal complex formation
Source: Genes Dev. 2020 Dec 1;34(23-24):1605–18. doi: 10.1101/gad.342873.120 (PMC7706706; doi:10.1101/gad.342873.120)
Supplement: Supplemental Material [file supp_gad.342873.120_Supplemental_material.pdf]

Supplemental material for:

Chromosome-autonomous feedback downregulates meiotic DNA break competence upon synaptonemal complex formation

Xiaojing Mu, Hajime Murakami, Neeman Mohibullah and Scott Keeney

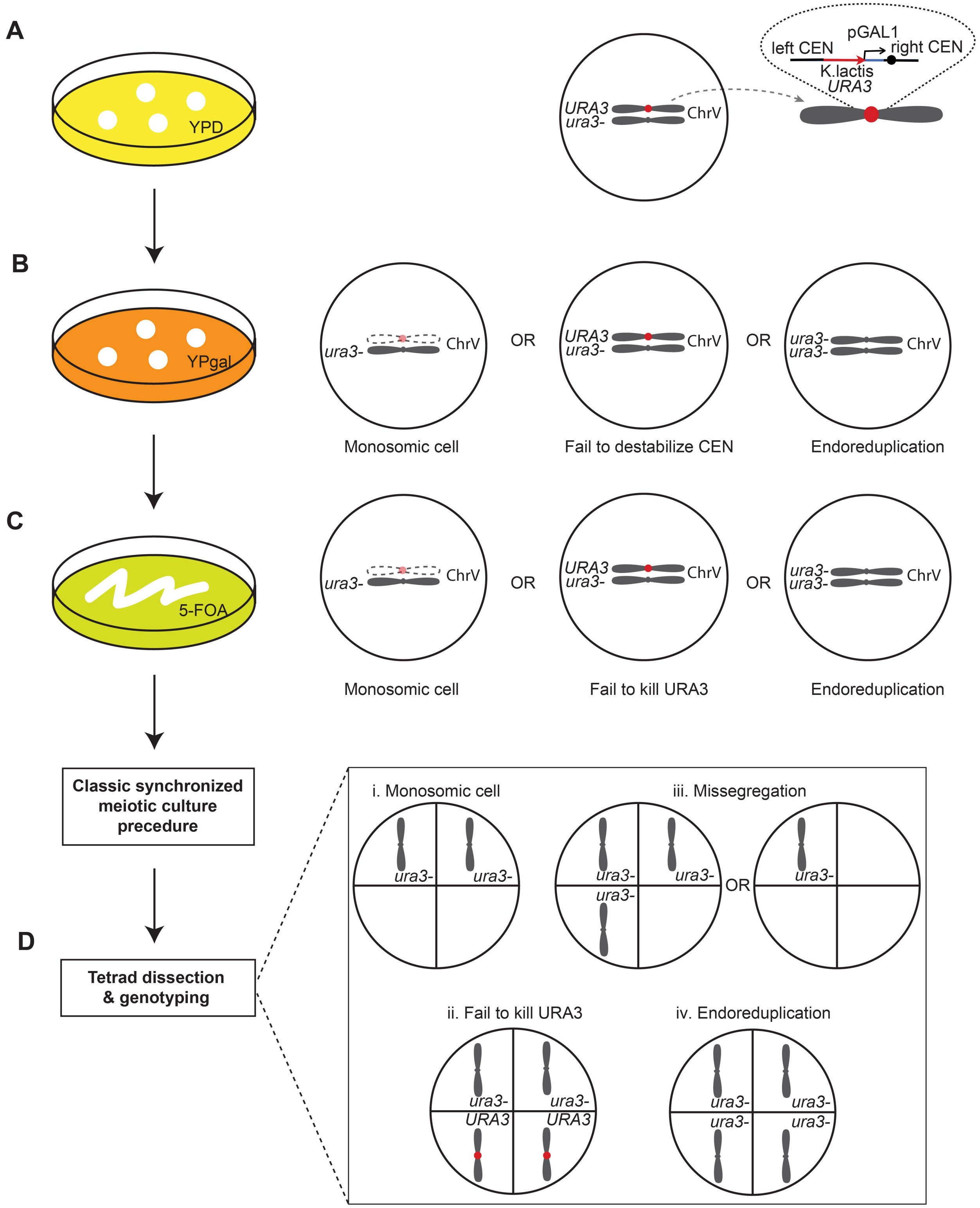

Supplemental Figure S1. Strategy for constructing the monosomic strain.
(A) In the diploid parental strain, one copy of chrV contains a conditional centromere generated by insertion of the *GAL1* promoter (marked with *URA3*) adjacent to the centromere. Cells are

cultivated on a YPD plate overnight before transferring to a YP galactose plate. (B) After 24 h galactose induction, most cells lose one copy of chrV due to the destabilized centromere. However, galactose induction may not be 100% efficient and some cells may have endoreduplicated the remaining copy, leading to a mixed population with different genotypes. (C) By patching cells on a 5-FOA plate, the majority of cells containing the *URA3* marker are killed, but a mixture of different cell types still potentially exists. (D) Tetrad dissection and spore genotyping after meiotic culturing are used to assess the population genotype as indicated (see **Supplemental Table S1**). i. Tetrads from monosomic cells should yield two Ura- spores and two dead spores lacking chrV. ii. Tetrads from cells that failed to destabilize the centromere and that escaped killing on 5-FOA should yield two Ura- and two Ura+ spores. iii. Chromosome missegregation in monosomic or endoreduplicated cells may yield tetrads with one or three Ura- spores, respectively. iv. Endoreduplication should yield tetrads with four Ura- spores.

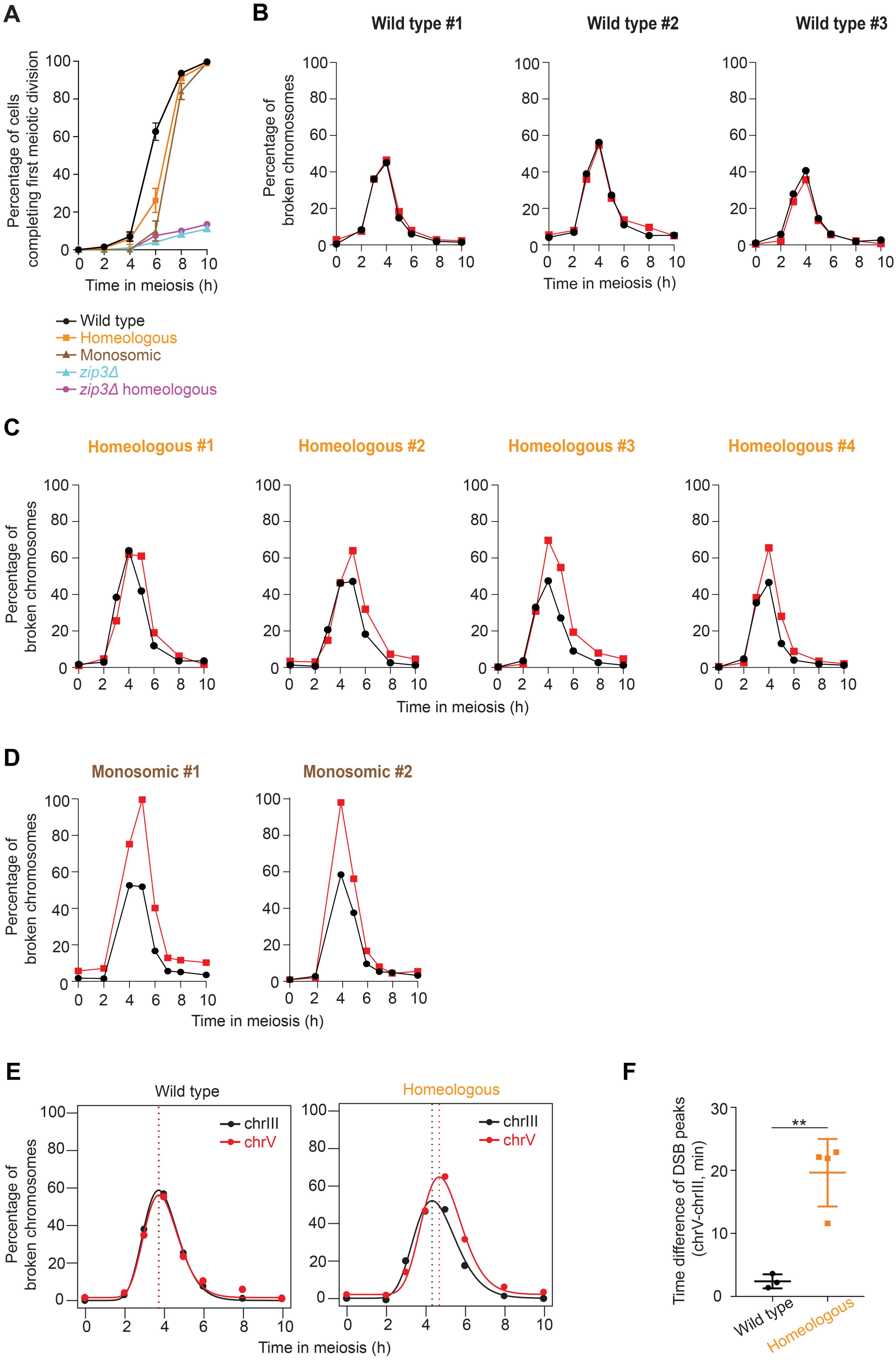

A
B
Wild type #1
Wild type #2
Wild type #3
Percentage of cells completing first meiotic division
Percentage of broken chromosomes
Time in meiosis (h)
Wild type
Homeologous
Monosomic
zip3Δ
zip3Δ homeologous
C
Homeologous #1
Homeologous #2
Homeologous #3
Homeologous #4
D
Monosomic #1
Monosomic #2
E
Wild type
Homeologous
chrIII
chrV
F
Time difference of DSB peaks (chrV-chrIII, min)
**
Wild type
Homeologous

Supplemental Figure S2. A later DSB peak on homolog-engagement-defective chromosomes.

(A) Meiotic division timing. The graph shows the percentage of cells that completed the first division (bi- and tetra-nucleate cells). ≥100 cells were counted at each time point for each sample. Error bars are mean ± SD for three wild type and four homeologous cultures; mean ± range for two each of monosomic, *zip3*Δ, and *zip3*Δ homeologous cultures. (B) Representative lognormal curves fitted to DSB time courses for chrIII and chrV in wild type and homeologous strains. (C) Quantification of DSB peak time differences after curve fitting between chrV and chrIII for wild type and homeologous strains. Each point is an independent culture; bars are mean ± SD. ** $p<0.01$, unpaired t test.

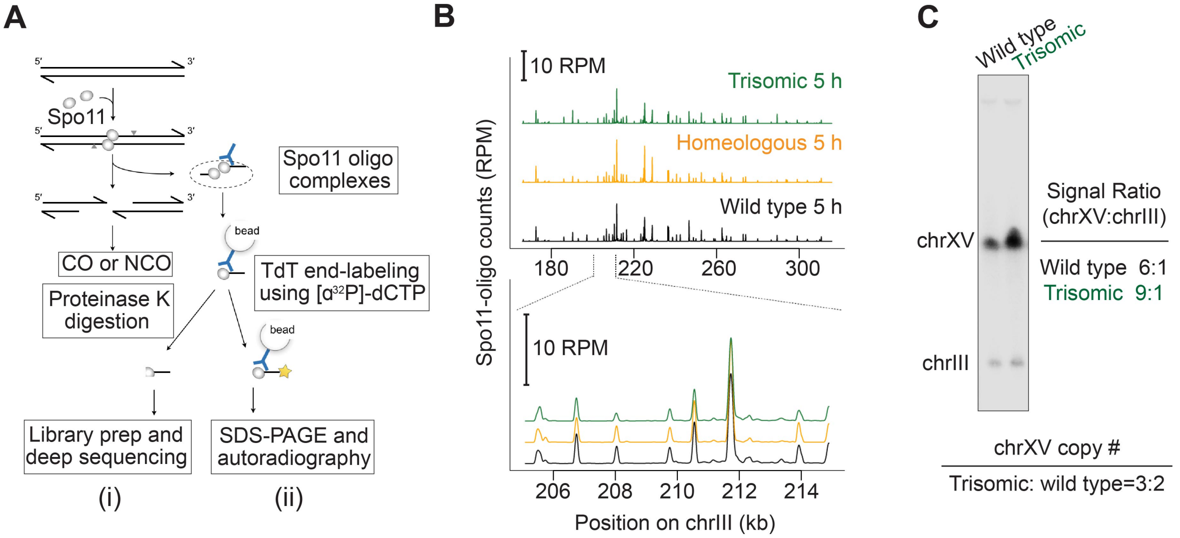

Supplemental Figure S3. DSB mapping and quantification via analysis of Spo11-oligo complexes, and karyotypic analysis of the trisomy-XV strain.
(A) Spo11 generates a covalent protein-linked DSB, then endonucleolytic cleavage releases Spo11 bound to short oligos. Spo11-oligo complexes can be detected by (i) Spo11-oligo mapping, in which immunoprecipitated Spo11-oligo complexes are digested with proteinase K and the released oligos are ligated to sequencing adaptors; or (ii) labeling of Spo11-oligo complexes, in which immunoprecipitated Spo11-oligo complexes are radiolabeled using terminal deoxynucleotidyl transferase and [α-^{32}P]-dCTP then separated by SDS-PAGE. (B) Spo11-oligo distributions on representative regions of chrIII. RPM, reads per million mapped; profiles were smoothed with a 201-bp Hann window. (C) Genotyping by Southern blotting to detect full-length chrXV and chrIII (control) separated by PFGE.

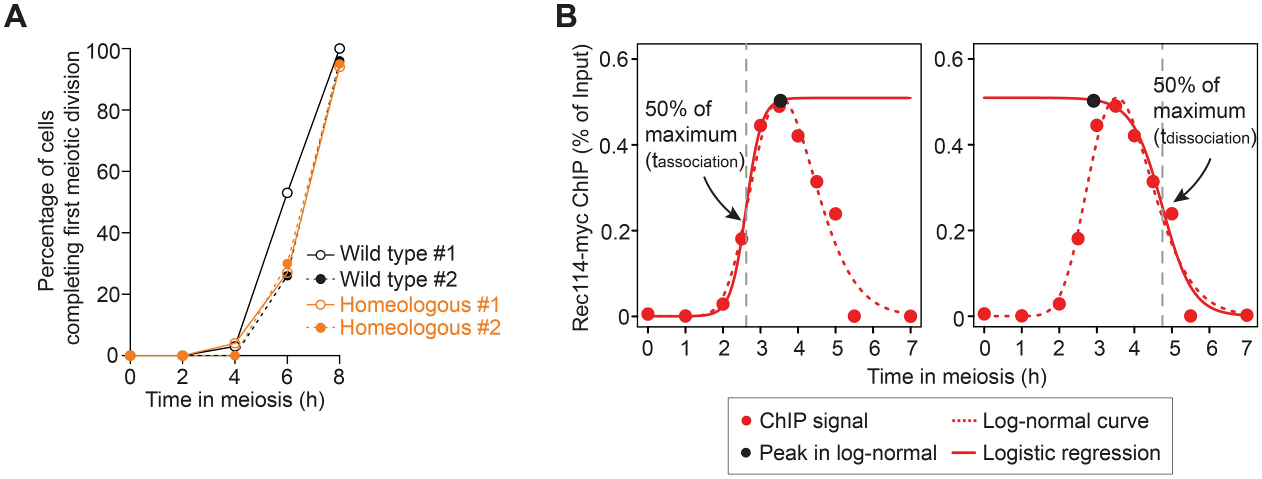

Supplemental Figure S4. Time course analysis of Rec114 binding on homeologous chrV. (A) Meiotic division timing. The graph shows the percentage of cells completing the first division (total bi- and tetra-nucleate cells) for individual cultures. ≥100 cells were counted at each time point for each sample. Note that wild type culture #2 appears slightly delayed. This exemplifies normal culture-to-culture variability in absolute timing of meiotic progression, highlighting the usefulness of internally controlled experiments. (B) Illustration of two-step curve fitting for calculations of association and dissociation times. A log-normal curve (dashed line) is first fitted to all points to define the peak time and height (black circle), then two logistic curves (solid lines) are fitted (left, using early points; right, using late points); $t_{association}$ is defined as the position where the logistic curve reaches 50% of the peak level for early points (left) and $t_{dissociaiton}$ is where the logistic curve reaches 50% of the peak level for late points (right).

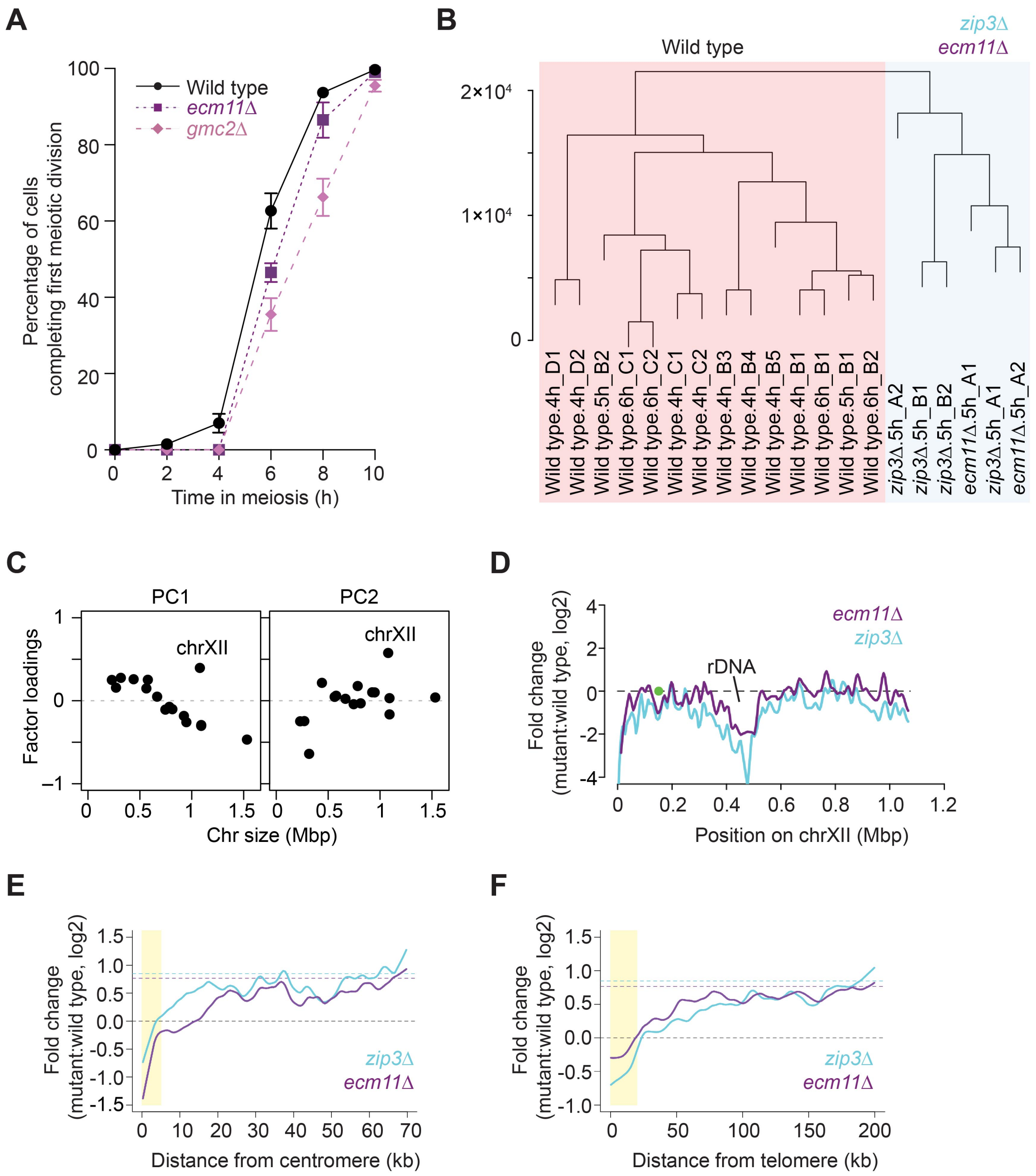

Supplemental Figure S5. DSB patterns in *gmc2*Δ and *ecm11*Δ mutants.
(A) Meiotic division timing. The graph shows the percentage of cells completing the first divisions (total bi- and tetra-nucleate cells, mean ± SD, 3 cultures each). ≥100 cells were counted at each time point for each culture. (B) Hierarchical clustering analysis using the "Ward D2" method for 20 wild-type and mutant maps. The y-axis represents the height of the cluster dendrogram. In the label for each data set, the letters A, B, C and D stand for maps generated by Xiaojing Mu, Neeman Mohibullah, Megan van Overbeek, and Xuan Zhu, respectively, and the numerals distinguish biological replicate maps. (C) Contributions (factor loadings) of each chromosome to PC1 and PC2 shown in **Fig. 6C**. PC1 appears to mainly capture effects of the mutations on chromosome size plus a contribution from the unique behavior of chrXII. The three shortest chromosomes and (in the opposite direction) chrXII were the greatest contributors to

PC2. The underlying source of this variation in PC2 is unclear, but as this dimension does not cleanly distinguish the data sets based on genotype (**Fig. 6C**), we speculate that PC2 is capturing culture-to-culture variability and/or experimental variability in sequencing library preparation. (D) Regional variation in response to *zip3*Δ and *ecm11*Δ mutations for chrXII. Lines, local regression (loess) based on Spo11-oligo density changes in 5 kb-bins; green circle, centromere; rDNA location is labeled. (E, F) Fold change of Spo11-oligo densities for *zip3*Δ and *ecm11*Δ compared to wild type in pericentric (E) and telomere-proximal (F) regions. Solid lines are local regression (loess) for data in 5 kb-bins averaged across 32 chromosome arms; dashed lines indicate no change in black and genome average change in cyan (1.8-fold, *zip3*Δ) and purple (1.7-fold, *ecm11*Δ); yellow shading highlights zones suppressed for DSB formation as previously defined (Pan et al. 2011).

Supplemental Table S1. Genotyping of monosomic biological replicates

Event	Observed phenotype	No. of viable spores	Culture[a]	
			A	B
Failure to kill *URA3* spores	2:2 Ura+:Ura-	4	0 (0/13)	0 (0/10)
Endoreduplication	Ura-	4	0 (0/13)	0 (0/10)
Monosomic chrV	Ura-	2	84.6% (11/13)	80% (8/10)
Missegregation	Ura-	1 or 3	15.4% (2/13)	20% (2/10)

[a] The number of dissected tetrads is indicated:

Culture A: 13 tetrads dissected in total

Culture B: 10 tetrads dissected in total

Supplemental Table S2. Primer pairs for ChIP-qPCR

Primer Name	Alias	Sequence	Chr	Position
PP1F	ARE1	GAAGATAGAGTTGCTGGTGAAAG	III	248737-248760
PP1R	ARE1	TAATTGTTACCGGGTGGGTC	III	248834-248853
PP2F	GAT1	TGTGTACTTAACCGTTTTCAGC	VI	89499-89521
PP2R	GAT1	CGAAAAATAGCAACCAGGCAC	VI	89488-89509
PP3F	GLY1	TGTCGTCGGTAGAACCTTG	V	82148-82166
PP3R	GLY1	TTCCTGGAGCGTTGGTAG	V	82261-82278
PP4F	Right arm 1	GATCCCGAACAAGTATCAGTCC	V	224319-224340
PP4R	Right arm 1	CGATGAGCTGGTCAAATCTG	V	224419-224438
PP5F	Right arm 2	AACTGCCACCTAATCGCTTC	V	531194-531213
PP5R	Right arm 2	TTCAAATGAAGCCAGGTCC	V	531236-531254

Supplemental Table S3. Yeast strains used in this study

Strain number	Species	Genotype
SKY4780	*S. cerevisiae* SK1	*MATa/α; ura3/"; lys2/"; ho::LYS2/"; leu2Δ(asp718-ecoR1)/leu2Δ; arg4Δ(eco47III-hpa1)/arg4Δ-bgl; cyh2-z/"; S. cerevisiae* chrV: *natMX/S. cerevisiae* chrV*; SPO11-6His-3FLAG-loxP-kanMX-loxP/"*
SKY4781	*S. cerevisiae* SK1	*MATa/α; ura3/"; lys2/"; ho::LYS2/"; leu2Δ(asp718-ecoR1)/leu2Δ; arg4Δ(eco47III-hpa1)/arg4Δ-bgl; cyh2-z/"; S. cerevisiae* chrV: *natMX/S. cerevisiae* chrV*; SPO11-6His-3FLAG-loxP-kanMX-loxP/"*
SKY4777[a]	*S. cerevisiae* SK1 w/ *S. pastorianus* chrV	*MATa/α; ura3/"; lys2/"; ho::LYS2/"; leu2Δ(asp718-ecoR1)/leu2Δ; arg4Δ(eco47III-hpa1)/arg4Δ-bgl; cyh2-z/"; S. pastorianus* chrV: *ilv1/S. cerevisiae* chrV: *natMX; SPO11-6His-3FLAG-loxP-kanMX-loxP/SPO11*
SKY4779[a]	*S. cerevisiae* SK1 w/ *S. pastorianus* chrV	*MATa/α; ura3/"; lys2/"; ho::LYS2/"; leu2Δ(asp718-ecoR1)/leu2Δ; arg4Δ(eco47III-hpa1)/arg4Δ-bgl; cyh2-z/"; S. pastorianus* chrV: *ilv1/S. cerevisiae* chrV: *natMX; SPO11-6His-3FLAG-loxP-kanMX-loxP/SPO11*
SKY4772[a]	*S. cerevisiae* SK1 w/ *S. pastorianus* chrV	*MATa/α; URA3/ura3; lys2/"; ho::LYS2/"; leu2Δ/"; arg4Δ/"; REC114-myc8::ura3::hphMX6/"; S. pastorianus* chrV: *ilv1/S. cerevisiae* chrV: *natMX*
SKY4773	*S. cerevisiae* SK1	*MATa/α; URA3/ura3; lys2/"; ho::LYS2/"; leu2Δ/"; arg4Δ/"; REC114-myc8::ura3::hphMX6/"; S. cerevisiae* chrV: *natMX/S. cerevisiae* chrV
SKY4774	*S. cerevisiae* SK1	*MATa/α; URA3/ura3; lys2/"; ho::LYS2/"; leu2Δ/"; arg4Δ/"; REC114-myc8::ura3::hphMX6/"; S. cerevisiae* chrV: *natMX/S. cerevisiae* chrV
SKY4776[a]	*S. cerevisiae* SK1 w/ *S. pastorianus* chrV	*MATa/α; ura3/"; lys2/"; ho::LYS2/"; leu2Δ/"; arg4Δ/"; REC114-myc8::ura3::hphMX6/"; S. pastorianus* chrV: *ilv1/S. cerevisiae* chrV: *natMX*
SKY5070[b]	*S. cerevisiae* SK1	*MATa/α; ura3/"; lys2/"; ho::LYS2/"; arg4Δ-bgl/"; leu2Δ/";GAL3/"; CEN5::pGAL1-CEN5-URA3/CEN5*
SKY5071[b]	*S. cerevisiae* SK1	*MATa/α; ura3/"; lys2/"; ho::LYS2/"; arg4Δ-bgl/"; leu2Δ/";GAL3/"; CEN5::pGAL1-CEN5-URA3/CEN5*

SKY5294[a]	*S. cerevisiae* SK1 w/ *S. pastorianus* chrV	*MATa/α; ura3/"; lys2/"; ho::LYS2/"; leu2Δ(asp718-ecoR1)/leu2Δ; arg4Δ(eco47III-hpa1)/arg4Δ-bgl; cyh2-z/"; S. pastorianus* chrV: *ilv1/S. cerevisiae* chrV: *natMX; SPO11-6His-3FLAG-loxP-kanMX-loxP/"*
SKY5325	*S. cerevisiae* SK1	*MATa; ura3; lys2; ho::LYS2; leu2Δ; arg4Δ-bgl; cyh2-z; zip3Δ:hphMX; SPO11-6His-3FLAG-loxP-kanMX-loxP*
SKY5327	*S. cerevisiae* SK1	*MATα; ura3; lys2; ho::LYS2; leu2Δ; arg4Δ-bgl; cyh2-z; zip3Δ::hphMX; SPO11-6His-3FLAG-loxP-kanMX-loxP*
SKY5469[a]	*S. cerevisiae* SK1 w/ *S. pastorianus* chrV	*MATa/α; ura3/"; lys2/"; ho::LYS2/"; leu2Δ/"; arg4Δ(eco47III-hpa1)/arg4Δ-bgl; zip3Δ::hphMX/"; S. pastorianus* chrV: *ilv1/S. cerevisiae* chrV: *natMX; SPO11-6His-3FLAG-loxP-kanMX-loxP/"*
SKY5642	*S. cerevisiae* SK1	*MATa/α; ura3/"; lys2/"; ho::LYS2/"; leu2Δ/"; arg4Δ-bgl/arg4Δ-nsp; cyh2-z/"; gmc2Δ::natMX/"; SPO11-6His-3FLAG-loxP-kanMX-loxP/"*
SKY5643	*S. cerevisiae* SK1	*MATa/α; ura3/"; lys2/"; ho::LYS2/"; leu2Δ/"; arg4Δ-bgl/arg4Δ-nsp; cyh2-z/"; gmc2Δ::natMX/"; SPO11-6His-3FLAG-loxP-kanMX-loxP/"*
SKY5869	*S. cerevisiae* SK1	*MATa/α; ura3/"; lys2/"; ho::LYS2/"; leu2Δ/"; arg4Δ-bgl/arg4Δ-nsp; cyh2-z/"; ecm11Δ::natMX/"; SPO11-6His-3FLAG-loxP-kanMX-loxP/"*
SKY5870	*S. cerevisiae* SK1	*MATa/α; ura3/"; lys2/"; ho::LYS2/"; leu2Δ/"; arg4Δ-bgl/arg4Δ-nsp; cyh2-z/"; ecm11Δ::natMX/"; SPO11-6His-3FLAG-loxP-kanMX-loxP/"*

[a] Haploid *S. cerevisiae* SK1 strain carrying *S. pastorianus* chrV from Michael Lichten

[b] Plasmid containing *CEN5::pGAL1-CEN5-URA3* from Rodney Rothstein

The trisomic chrXV strain arose spontaneously during normal culturing

Supplemental Table S4. Mapping statistics for Spo11 oligo sequences

Dataset	Strain	Time	No. of total reads	Genome mapped to	No. mapped	No. mapped uniquely
Homeo_1[a]	SKY5294	4 h	19,996,390	*S. cerevisiae* S288C plus *S. eubayanus* chromosome V	17,815,499	15,000,784(98.1% of filtered)
Homeo_2[a]	SKY5294	5 h	33,244,263	*S. cerevisiae* S288C plus *S. eubayanus* chromosome V	30,836,597	25,837,596(97.2% of filtered)
*zip3*Δ_1[b]	SKY5325 x SKY5327	5 h	34,251,244	*S. cerevisiae* S288C	33,274,600	28,128,137(98.1% of filtered)
*zip3*Δ_2[b]	SKY5325 x SKY5327	5 h	77,343,014	*S. cerevisiae* S288C	75,329,644	67,157,785(98.1% of filtered)
*zip3*Δ homeo_1[a]	SKY5469	5 h	90,075,914	*S. cerevisiae* S288C plus *S. eubayanus* chromosome V	16,625,735	13,928,149(98.0% of filtered)
*zip3*Δ homeo_2[a]	SKY5469	5 h	7,218,891	*S. cerevisiae* S288C plus *S. eubayanus* chromosome V	3,796,498	3,356,563 (97.1% of filtered)
*ecm11*Δ_1[b,*]	SKY5869	5 h	10,778,802	*S. cerevisiae* S288C	10,648,118	1,435,809 (98.0% of filtered)
*ecm11*Δ_2[b]	SKY5869	5 h	9,679,255	*S. cerevisiae* S288C	8,086,230	7,254,361 (98.0% of filtered)

[a] One copy of chrV is from *S. pastorianus* and all other chromosomes are from the *S. cerevisiae* SK1 strain. Sequencing reads were mapped to the S288C genome (sacCer2 assembly) plus *S. eubayanus* chrV.

[b] Strains are *S. cerevisiae* SK1 background, mapped to the sacCer2 assembly.

[*] For this sample, a synthetic oligonucleotide (TGGATCCGTGGAATAATGCACAATT) was added to increase the DNA amount during library preparation.

Supplemental Table S5. Spo11-oligo datasets used in this study

Source (accession number)	Dataset as shown at GEO	Alias used in Supplemental Figure S5	Time	Tag	Figures
**Thacker et al. 2014**	WT_1	Wild type.4h_B3	4 h	PrA	3, 4 and 6; S5
**(GSE48299)**	WT_2	Wild type.4h_B4	4 h	PrA	3, 4 and 6; S5
	zip3_1	zip3Δ.5h_B1	5 h	PrA	6; S5
	zip3_2	zip3Δ.5h_B2	5 h	PrA	6; S5
**Mohibullah**	wt4_1	Wild type.4h_B1	4 h	PrA	2 and 6; S5
**and Keeney 2017**	wt4_2	Wild type.4h_B5	4 h	PrA	6; S5
**(GSE84696)**	wt5_1	Wild type.5h_B1	5 h	PrA	2 and 6; S3 and S5
	wt5_2	Wild type.5h_B2	5 h	PrA	6; S5
	wt6_1	Wild type.6h_B1	6 h	PrA	2 and 6; S5
	wt6_2	Wild type.6h_B2	6 h	PrA	6; S5
**Murakami et al. 2020**	wt_a_4h	Wild type.4h_C1	4 h	flag	6; S5
**(GSE119689)**	wt_b_4h	Wild type.4h_C2	4 h	flag	6; S5
	wt_a_6h	Wild type.6h_C1	6 h	flag	6; S5
	wt_b_6h	Wild type.6h_C2	6 h	flag	6; S5

**Zhu and Keeney 2015**	WT_1	Wild type.4h_D1	4 h	flag	6; S5
**(GSE67910)**	WT_2	Wild type.4h_D2	4 h	flag	6; S5
**This study**	SK1_homeologous_4h		4 h	flag	2 and 6
**(GSE152957)**	SK1_homeologous_5h		5 h	flag	2, 3 and 6; S3
	SK1_zip3Δ_1	*zip3*Δ.5h_A1	5 h	flag	4 and 6; S5
	SK1_zip3Δ_2	*zip3*Δ.5h_A2	4 h	flag	4 and 6; S5
	SK1_zip3Δ homeo_1		5 h	flag	4 and 6
	SK1_zip3Δ homeo_2		5 h	flag	4 and 6
	SK1_ecm11Δ_1	*ecm11*Δ.5h_A1	5 h	flag	6; S5
	SK1_ecm11Δ_2	*ecm11*Δ.5h_A2	5 h	flag	6; S5
	SK1_trisomic_4h		4 h	PrA	2
	SK1_trisomic_5h		5 h	PrA	2 and 3; S3
	SK1_trisomic_6h		6 h	PrA	2
